# Chronic corticosterone deteriorates latrine and nesting behaviours in mice

**DOI:** 10.1098/rsos.220718

**Published:** 2023-02-01

**Authors:** Kensaku Nomoto, Kenji Kansaku

**Affiliations:** Department of Physiology, Dokkyo Medical University School of Medicine, Mibu 321-0293, Tochigi, Japan

**Keywords:** chronic corticosterone, self-care, self-neglect, latrine, defecation, laboratory mice

## Abstract

Self-care behaviours are actions that help maintain good health and surroundings. For example, appropriate toileting, sleeping in the bed, and bathing and washing are among self-care behaviours in humans. Animals also perform similar self-care behaviours such as latrine, nesting and self-grooming. Studies have shown that chronic stress disrupts nesting and self-grooming behaviours. However, the effect of chronic stress on latrine behaviour, preferential, repeated defecation at specific locations, has not yet been clarified. This study aimed to investigate the influence of chronic corticosterone administration on latrine and nesting behaviours in mice. The variation in defecation location was quantified as the degree of the latrine behaviour by using Shannon entropy. The nest quality was scored based on shape. The study showed that mice exposed to chronic corticosterone had scattered defecation sites and lower nest quality compared to the control group. Furthermore, results showed that more scattered defecation behaviour was associated with lower nest quality at an individual level. Additionally, the deterioration of these self-care behaviours was associated with depression-like behaviours such as less open field activity and increased immobility time during the tail suspension test. These results suggest that chronic corticosterone deteriorates self-care behaviours such as latrine and nesting in mice.

## 1. Introduction

Self-care behaviour is defined as the actions that individuals take to maintain health and hygiene [1]. For example, appropriate toileting, sleeping in the bed, and bathing and washing are among self-care behaviours in humans. The concept of self-care has been a topic in the fields of nursing sciences, veterinary medicine and animal welfare [2–5]. By contrast, disruption of self-care behaviour can cause harm to one's health and hygiene, which is called self-neglect [6,7].

Animals also perform similar self-care behaviours such as latrine, nesting and self-grooming behaviours. Latrine behaviour refers to preferential, repeated defecation at specific locations [8]. It is observed among a wide range of animal species, from birds to mammals [8–13]. Laboratory mice are known to defecate at a specific location in their home cages when housed in groups of mice that are left undisturbed [14,15]. Latrine behaviour is beneficial in preventing infectious diseases [12]. Nesting and self-grooming behaviours are also widespread. Mice tend to build dome-shaped nests with bedding materials [16,17], and nesting behaviour is considered useful for thermoregulation [16]. Self-grooming behaviour is an innate behaviour which is helpful for hygiene maintenance and thermoregulation [18]. These behaviours promote one's health and improve environmental and personal hygiene, and thus are considered self-care behaviours.

Previous studies have shown that chronic stress can impair self-care behaviours such as nesting and self-grooming behaviours. Subchronic and mild social defeat stress induces temporary deficits in nesting behaviour [19]. Chronic restraint stress also impairs nesting behaviour [20]. Self-grooming behaviour was impaired by chronic stress such as chronic mild stress [21] and four-week corticosterone exposure [22]. In contrast with nesting and self-grooming behaviours, the effects of chronic stress on latrine behaviour have been understudied.

To clarify this issue, this study evaluated whether the chronic administration of corticosterone disrupts latrine and nesting behaviours in mice. Chronic administration of corticosterone, the main glucocorticoid in rodents, was used as a means of providing chronic stress [23,24]. While one group of mice received oral administration of corticosterone (CORT group) for at least three weeks, the other group was administered with vehicle solution (VEH group). The qualities of latrine and nesting behaviours were evaluated once a week based on the variation in defecation location and the shape of a nest, respectively. In so doing, the present study provided the first experimental evidence that chronic corticosterone deteriorates latrine and nesting behaviours in mice.

## 2. Methods

### 2.1. Animals

The data used in this study was from male C57BL/6NCrl purchased at seven weeks of age from Charles River Japan (*N* = 42). The mice were housed in groups of 4–5 upon arrival and acclimatized to the laboratory for at least one week; the light cycle was 12 : 12 h (lights on at 07 : 00), and food and water were provided ad libitum. The ambient temperature was conditioned at 23 ± 1°C. All procedures were approved by the Ethics Committee for Animal Experiments of Dokkyo Medical University (Approval No. #1341). During the experiment, one mouse died (CORT group) and the data of three mice were unreliable due to technical problems such as water leakage (two mice from the CORT group; one mouse from the VEH group); thus, they were excluded from subsequent analyses. Statistical calculations were not used to determine the sample size. The sample size was determined using standards in the field established in previous published studies.

### 2.2. Experimental design

After the start of the experiment, mice were housed individually in small home cages (size, 140 × 320 × 140 mm, Natsume Seisakujo, Japan). Paper-like white bedding was used (Paperclean, SLC Japan). The mice were randomly assigned to CORT and VEH groups. While the mice of the CORT group were administered with corticosterone (Fuji Wako Chemical, Japan) dissolved in 1% ethanol solution (Fuji Wako Chemical, Japan) to a final concentration of 0.1 mg ml^−1^ (CORT, *N* = 18), the mice of the VEH group were administered with 1% ethanol solution (VEH, *N* = 20) through drinking water for three weeks [25]. In previous studies, the duration of corticosterone exposure varied from three weeks [25], four weeks [22,26], to five weeks or more [24]. In order to validate our behavioural assay with longer exposure duration, of 38 animals, 18 (CORT, *N* = 9; VEH, *N* = 9) received an additional week of exposure for a total of four weeks. At the end of the exposure period, mice underwent an open field test (OFT), sucrose preference test (SPT) and tail suspension test (TST). The behavioural tests were videotaped for offline analysis. Furthermore, body weight, water intake and food intake were measured during weekly cage changes.

### 2.3. Evaluation of latrine behaviour

The faecal clutter was quantified weekly. During weekly cage changes, the mouse was gently removed from a home cage. Photos were taken of the bottom of the home cage. The following imaging processing was performed by a custom-made MATLAB script. First, the photo was binarized using adaptive thresholding (adaptthresh with a sensitivity of 0.1). Thereafter, a morphological filter was applied to eliminate salt and pepper noise (imopen and imclose by rectangle structural elements with a size of 5 pixels). The black faeces contrasted well against the white paper bedding. Thereafter, a two-dimensional histogram of faecal frequency was created by binning with a square of approximately 1 cm in size. For this faecal frequency image, Shannon entropy was calculated (entropy in Image Processing Toolbox) and its value was defined as a clutter index. Since the faecal frequency image has 256 (= 2^8^) greyscale values, the maximum value of the clutter index was 8. If the mice defecate in a certain place, the defecation frequency will be high only in that bin, and relatively low in the other bins. Therefore, the frequency distribution will not be even and the clutter index will remain low. By contrast, if the mice defecate in scattered locations, the frequency distribution will be closer to a uniform distribution and the clutter index will be higher. We chose to interpret a low clutter index as appropriate latrine behaviour in this study. Because this analysis was computerized and unbiased, blinded assessment was not used.

### 2.4. Evaluation of nesting behaviour

Nest scoring was conducted on the last day of corticosterone administration. At moderate temperature, mice are known to build fluffy, dome-shaped nests when supplied with appropriate nesting materials [16,17]. The nest site was identified as the resting place for the mouse during the light period before the cage change. The shape of the nest was visually evaluated based on its height. Modified from the previous study [17], a photo of the nest from the side of the home cage was taken and scored on a 5-point scale: 5, complete dome-shaped nest; 4, incomplete dome-shaped nest; 3, cup nest; 2, flat nest; 1, the nesting materials were manipulated, but not gathered; 0, the nesting materials were not manipulated. Since we did not use additional nesting materials, scores 0 and 1 were not applicable in this study. Every week, the cages were changed and new bedding was added. Thus, this index reflects nesting behaviour over a week. A high nesting score indicates good self-care behaviour. One mouse from the VEH group was excluded because the photo of the nest of this animal was not recorded due to human error. The photos of the nest were anonymized, and the analysis was performed blindly.

### 2.5. Other behavioural tests

In addition, other behavioural tests were performed after the corticosterone exposure.

#### 2.5.1. Open field test

The OFT was performed at 15 : 00–17 : 00 on the day the exposure ended. Mice were allowed to acclimate to the experimental room for at least 30 min before the test. Mice were placed in a corner of an arena (size, 450 × 450 × 450 mm) and observed for 5 min. The central zone was defined as a 225 × 225 mm area in the centre of the arena. The offline video analysis was used to analyse the time spent in the centre and the total distance travelled [27]. Because the analysis was computerized and unbiased, blinded assessment was not used. After the behavioural test, the number of faecal pellets in the arena was counted.

#### 2.5.2. Sucrose preference test

The SPT was started from 17 : 30 to 18 : 30 on the day of exposure termination. Two bottles of tap water and 1% sucrose solution were given in the home cage. The mice had never been provided with sucrose solution before the test. Food and water were available ad libitum before and during the test. The weight of the bottles was measured before the test. A bottle containing tap water was placed where a bottle had been placed during the exposure period, and a bottle containing 1% sucrose solution was placed next to the bottle of tap water. The locations of the bottles were kept in their original positions throughout the SPT. Mice were free to drink water from any of the two bottles according to their preference. At 11 : 30 on the next day, the weight of the bottles was measured, and the amount of liquid consumed by the mouse was calculated. The sucrose preference index was defined as the amount of sucrose water drunk divided by the sum of the amount of liquid consumed. When the sucrose preference index is 1.0, a mouse exclusively prefers sucrose water. When the sucrose preference index is 0.5, a mouse drinks tap and sucrose water in equal proportions. When the sucrose preference index is 0.0, a mouse exclusively prefers tap water. Blinded assessment was not performed.

#### 2.5.3. Tail suspension test

The TST was performed at 15 : 00–17 : 00 on the day after the end of the exposure. Mice were acclimated to the experimental room for at least 30 min before the test. Mending tape was applied approximately 2 cm from the tail end of the mouse, the tape was fixed to the horizontal bar of the behavioural apparatus, and the mouse was suspended for 6 min [28]. The behavioural experiment was recorded and the custom-made Bonsai workflow was used to annotate the moving time and calculate the immobility time [27]. The behavioural videos were anonymized, and the analysis was performed blindly.

### 2.6. Faecal corticosterone and corticosterone metabolites assay

For 20 mice (CORT, *N* = 9; VEH, *N* = 11), faecal corticosterone and corticosterone metabolites levels were measured after three-week exposure of corticosterone. The bedding was changed on day 21 and the next day's bedding was taken to collect faeces. The faeces were weighted and stored in a −20°C freezer until the assay. Corticosterone and corticosterone metabolites levels were measured using a commercially available ELISA kit (Corticosterone ELISA kit, Item No. 501320; Cayman Chemicals, Ann Arbor, MI, USA). The extraction of faecal corticosterone from the samples was done according to the manufacturer's instructions. Briefly, the faeces were thawed, dried in a heating block at 70–80°C and crushed into powder. We placed 25 mg of dry faeces into clean tubes and added 500 µl of 80% methanol. The tubes were shaken for 1 h and centrifuged at 10 000 × g for 10 min. Then, 400 µl of supernatant was transferred to a new clean tube, and methanol was evaporated in a heating block at 70–75°C. The samples were suspended in 500 µl of ELISA buffer. Thereafter, 50 µl of standards and samples were added in duplicate and in triplicate, respectively, to wells of a plate included in the kit. We added 100 µl and 50 µl of ELISA buffer to NSB and B_0_ wells, respectively. Then, 50 µl of corticosterone AChE tracer (except total activity and blank wells) and 50 µl of corticosterone ELISA antiserum (except NSB, total activity and blank wells) were added to each well, and the plate was incubated overnight at 4°C. After emptying the wells and rinsing five times with wash buffer, 200 µl of Ellman's reagent was added to each well and 5 µl of AChE tracer was added to the total activity well. The plate was incubated for 60–120 min in the dark on a shaker and read at 412 nm using a plate reader. Corticosterone levels were calculated according to the standard curve. Blinded assessment was not performed.

### 2.7. Data analysis

MATLAB 2020b (Mathworks, Natick, MA, USA) and GraphPad Prism 8 (GraphPad Software, San Diego, CA, USA) were used for data analysis and statistical analysis. The significance level was set at 0.05.

## 3. Results

This study examined whether latrine and nesting behaviours were impaired by chronic corticosterone administration in mice. During the experiment, self-care behaviours such as latrine and nesting behaviours were evaluated once a week among mice housed individually. For 20 mice (CORT, *N* = 9; VEH, *N* = 11), faecal corticosterone levels were measured after chronic corticosterone exposure. As expected, faecal corticosterone levels were higher in the CORT group than in the VEH group, validating the method of corticosterone administration (electronic supplementary material, figure S1).

**Figure 1.**
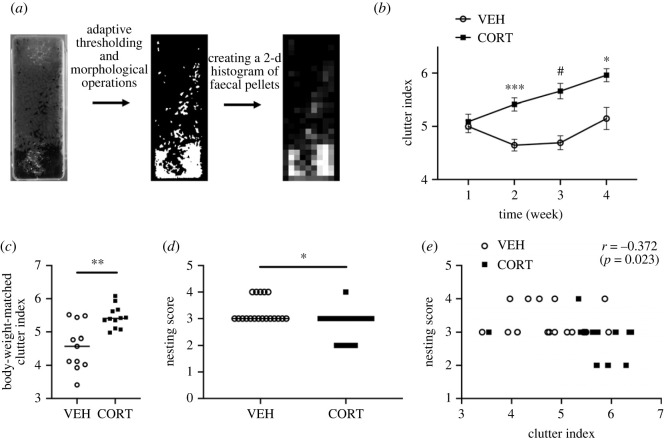
Chronic corticosterone disrupts latrine and nesting behaviours in mice. Filled squares indicate the CORT group; open circles indicate the VEH group. (*a*) Calculation of the clutter index. A photograph of the floor of a cage (left) was binarized (middle), which was further binned with a square of approximately 1 cm in size (right), showing the two-dimensional occurrence distribution of faecal pellets. The clutter index was defined as the Shannon entropy of this image. (*b*) The CORT group defecates in a more cluttered manner. The clutter index of the CORT group was higher than that of the VEH group (week 1–3, *N* = 18 and 20 for CORT and VEH groups, respectively; week 4, *N* = 9 and 11 for CORT and VEH groups, respectively; mixed-effects analysis was performed by constructing a linear mixed model with treatment and time as fixed effects and subject as a random effect; main effect of treatment, *p* < 0.0001, *F*_1,36_ = 24.78; main effect of time, *p* = 0.0138, *F*_2.519,73.88_ = 4.091; interaction effect, *p* = 0.0003, *F*_3,88_ = 6.896; *post hoc* Sidak multiple comparison test, week 1, *p* = 0.9787, week 2, *p* = 0.0002, week 3, *p* < 0.0001, week 4, *p* = 0.0205). Error bars represent s.e.m. (*c*) Body weight-matched clutter index. The clutter index was higher in the CORT group than in the VEH group even when we matched the body weight, comparing the CORT group after two weeks and the VEH group after three weeks (*N* = 12 and 11 for CORT and VEH groups, respectively; Mann–Whitney test, *p* = 0.0086, *U* = 24). (*d*) The CORT group makes a low-quality nest (two-way ANOVA, main effect of treatment, *p* = 0.0051, *F*_1, 33_ = 8.989; main effect of time, *p* = 0.2223, *F*_1,33_ = 1.547; interaction effect, *p* = 0.9312, *F*_1,33_ = 0.007562). Treatment-wise comparisons were shown for display purposes. Statistical analysis was performed by two-way ANOVA with the factors of treatment (CORT/VEH) and time (three week/four week) (see also electronic supplementary material, figure S2). (*e*) Correlation between the clutter index and the nesting score (Pearson correlation test, *r* = −0.372, *p* = 0.023). *p < 0.05, **p < 0.01, ***p < 0.001, ^#^*p* < 0.0001. See also electronic supplementary material, figure S1.

First, the latrine behaviour was analysed using Shannon entropy to quantify the variability in defecation sites. Since it has been reported that laboratory mice tend to defecate in a specific location in their home cage to keep their environment clean [14,15], scatteredness in defecation sites represents the quality of latrine behaviour. During the weekly cage changes, photos of the bottom of the home cage were taken (figure 1*a*, left) and the faeces site was extracted using image processing (figure 1*a*, middle). As a result, a two-dimensional histogram of the frequency of faecal locations was created (figure 1*a*, right). The Shannon entropy of this image is referred to as the clutter index. The clutter index is low when mice demonstrate appropriate latrine behaviour by defecating in a certain place, whereas the clutter index increases when mice defecate in scattered places, indicating inadequate latrine behaviour. The clutter indices between the CORT and VEH groups did not differ after one week. However, the clutter index of the CORT group was significantly greater than that of the VEH group (figure 1*b*) after two weeks, suggesting that the CORT group did not perform appropriate latrine behaviour.

The body weight and weight of faeces were significantly higher in the CORT group than in the VEH group (electronic supplementary material, figure S1), and there was a significantly high correlation between body weight and faeces quantity (Pearson correlation test, *r* = 0.6469, *p* = 0.0021). The CORT group also had a higher food intake and water consumption (electronic supplementary material, figure S1). Arguably, the increased clutter index of the CORT group can be attributed to heavy body weight and large faeces quantities. To test this argument, the clutter index of the CORT group after two weeks of corticosterone administration was compared with that of the VEH group after three weeks of administration, which showed similar body weight. The data from mice whose body weights were between 27 g and 30 g were used for the analysis. Results showed that the clutter index of the CORT group was significantly higher than that of the VEH group, even in weight-controlled mice (figure 1*c*). This suggests that the high clutter index of the CORT group cannot be explained solely by body weight and weight of faeces.

Subsequently, nesting behaviours were analysed. Mice are known to build fluffy dome-shaped nests when supplied with appropriate nesting materials [16,17]. Although additional nesting materials were not provided in this study, the mice were able to build a nest using paper bedding materials that were loosened and piled up. The nest site was identified as the resting place of a mouse during the light period before the cage change. Furthermore, the nest shape was evaluated across a 5-point scale based on height criteria modified from a previous study [17]. The score reported in this study reflects nesting behaviour over a week. The results showed that nest quality was lower in the CORT group than in the VEH group (figure 1*d*).

This study showed that corticosterone administration resulted in increased scattered faecal locations and reduced nest quality. To anchor the results on latrine behaviour in the context of self-care behaviours, the correlation between latrine behaviour and nesting behaviour, a typical self-care behaviour, was examined. There was a significant negative correlation between the clutter index and the nesting score (figure 1*e*). This indicates that individual mice with scattered defecation sites have low-quality nests. These results suggest that corticosterone administration degraded the self-care behaviours such as latrine and nesting behaviours.

**Figure 2.**
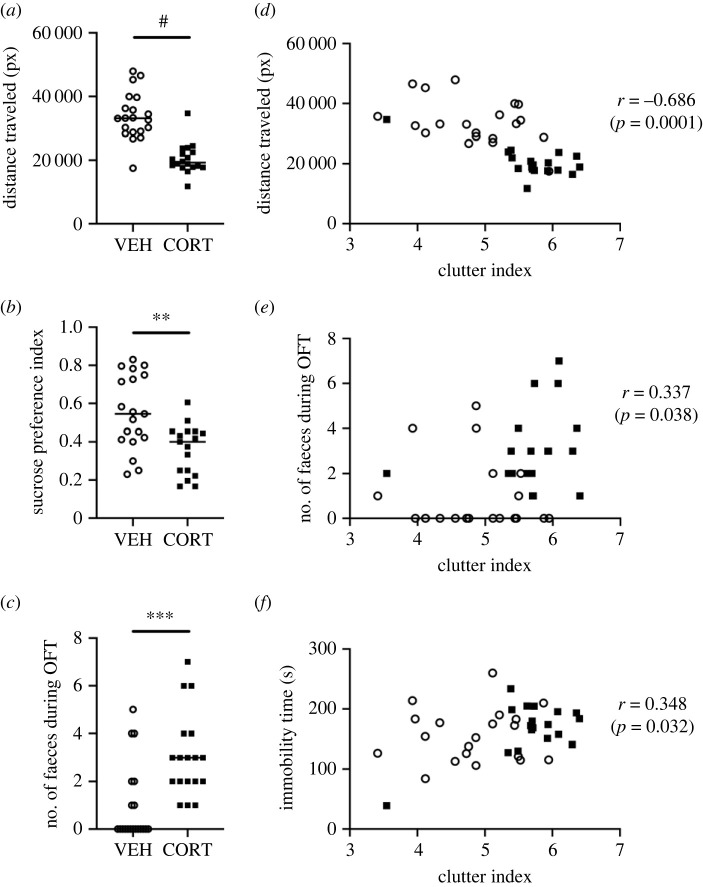
Chronic corticosterone induces depressive-like behaviour and depressive-like behaviour is correlated with the degree of deterioration of latrine behaviour. Treatment-wise comparisons were shown for display purposes. Statistical analysis was performed by two-way ANOVA with the factors of treatment (CORT/VEH) and time (three week/four week) (see also electronic supplementary material, figure S2). Filled squares indicate the CORT group; open circles indicate the VEH group. (*a*) Total moving distance during the OFT. The CORT group moved less than the VEH group (two-way ANOVA, main effect of treatment, *p* < 0.0001, *F*_1, 34_ = 49.13; main effect of time, *p* = 0.0088, *F*_1, 34_ = 7.733; interaction effect, *p* = 0.1743, *F*_1, 34_ = 1.925). (*b*) The sucrose preference index was smaller in the CORT group (two-way ANOVA, main effect of treatment, *p* = 0.0022, *F*_1,32_ = 11.04; main effect of time, *p* = 0.5292, *F*_1,32_ = 0.4047; interaction effect, *p* = 0.8116, *F*_1, 32_ = 0.05777). (*c*) The number of faecal pellets during the OFT. The CORT group defecated more (two-way ANOVA, main effect of treatment, *p* = 0.0004, *F*_1, 34_ = 15.38; main effect of time, *p* = 0.0325, *F*_1, 34_ = 4.973; interaction effect, *p* = 0.6071, *F*_1,34_ = 0.2694). (*d*) There was a significant negative correlation between the clutter index and the distance travelled in the OFT (Pearson correlation test, *r* = −0.686, *p* < 0.0001). (*e*) There was a significant positive correlation between the clutter index and the number of faeces during the OFT (Pearson correlation test, *r* = 0.337, *p* = 0.038). (*f*) There was a significant positive correlation between the clutter index and the immobility time in the TST (Pearson correlation test, *r* = 0.348, *p* = 0.032). *p < 0.05, **p < 0.01, ***p < 0.001, ^#^*p* < 0.0001.

Studies have shown that chronic corticosterone can cause depressive- and anxiety-like behaviours [22–24]. Consequently, after the completion of corticosterone exposure, this study performed behavioural tests for depressive- and anxiety-like behaviours, namely, OFT, SPT and TST. We predicted that mice exhibited depressive- and anxiety-like behaviours in response to chronic stress. In accordance with previous studies, the CORT group exhibited depressive-like behaviour such as low activity in the OFT (figure 2*a*) and low-sucrose preference in the SPT (figure 2*b*). In addition, increased defecation was observed in the CORT group during the OFT (figure 2*c*). However, there were no significant differences observed in the time spent in the central zone of the arena during the OFT and the immobility time during the TST (electronic supplementary material, figure S2). Thereafter, the relationship between self-care behaviours and depressive- and anxiety-like behaviours was examined. The correlation analysis showed that individual mice with high clutter index exhibited less OFT activity (figure 2*d*), an increased number of faecal pellets during the OFT (figure 2*e*) and longer periods of immobility during the TST (figure 2*f*). These results suggest that chronic corticosterone increased depressive-like behaviours, which correlated with the degree of deterioration in self-care behaviours.

## 4. Discussion

This study evaluated whether chronic corticosterone administration disrupts latrine and nesting behaviours. We found that chronic administration of corticosterone resulted in cluttered defecation sites and the deterioration of nest quality. These results suggest that chronic corticosterone worsens self-care behaviours such as latrine and nesting behaviours. Furthermore, the deterioration in self-care behaviour was associated with an increase in depressive-like behaviour.

The study shows that chronic corticosterone exposure disrupts latrine behaviour. Defecation at a specific location is common among a wide range of animal species [8–13]. Laboratory mice are also known to defecate at a specific location in their home cages [14,15]. This behaviour has ecological significance in terms of disease prevention and predator avoidance [11,12]. However, this variation in defecation sites has not been reported in-depth. Strong emotional stimuli such as fear are known to induce defecation behaviours [29]. The mice in this experiment were housed in their home cages without any exposure to external stimuli that can induce strong emotion; thus, it is unlikely that cluttered defecation in this study was caused by strong emotion. Another previous study reported that chronic social defeat stress could increase gastrointestinal motility [30]. It remains unclear whether the variation in the location of defecation was due to the inability to reach the location of defecation (i.e. faecal incontinence) or a lack of motivation to defecate at the specific location. The clutter index of CORT and VEH groups significantly differed after two weeks of corticosterone administration, suggesting that the administration of corticosterone for this duration was sufficient in disrupting latrine behaviour.

This study also shows that chronic administration of corticosterone deteriorates the quality of nests. It has been reported that social stress can induce temporary deficits in nesting behaviour [19]. A previous study reported that mice did not build nests in the first 7 h after 10 days of mild social defeat stress. However, the stressed mice were able to build comparable nests to those of the control after 23 h. In the present study, nesting behaviour was assessed once a week, which means that the degradation of nesting behaviour in the CORT group persisted for a week. This difference may be due to the severity of stress or to the difference in the modality of stressors. Nesting behaviour is disrupted by a variety of factors, including physical stresses such as surgery, pain, inflammation and animal models of neuropsychiatric disorders [2,5]. We are yet to clarify whether disrupted defecation behaviour is observed in these animal models. The present study found no significant differences in the quality of nests between three-week and four-week corticosterone administration groups. In conjunction with the results regarding latrine behaviour, this suggests that three-week corticosterone administration is sufficient in self-care behaviours such as latrine and nesting.

The present results showed that corticosterone exposure resulted in increased depressive-like behaviour, which is consistent with previous studies [23,24]. In contrast with previous findings, we failed to observe significant differences in some behavioural indices such as immobility time in the TST and the time staying in the central zone of the area in the OFT. A previous study has shown that differences in the methods such as the sex, the strain of mice, the administration method and the dose of corticosterone, and the housing condition can affect the behavioural results [31]. Accordingly, the discrepancy between our and previous studies could be explained by the fact that some of VEH mice could be stressed by isolation procedures. The caveat in behavioural tests is that, since we did not counterbalance the positions of the bottles in the SPT, a decrease in the sucrose preference index in the CORT group might be confounded with effects of neophobia. Furthermore, this study showed that the clutter index was correlated with several behavioural indices of depressive-like behaviour, such as activity on the OFT, faecal quantity during the OFT and immobility time on the TST. This indicates that individual mice with degraded self-care behaviours exhibit increased depression-like behaviours, suggesting an association between depression and disruption of self-care behaviours. As reported previously, depression is one of the risk factors for self-neglect [6,7]. Interestingly, patients with major depressive disorder have been associated with an abnormal glucocorticoid system resulting in elevated plasma cortisol levels [32,33]. Thus, an abnormal cortisol level that is accompanied by depression may induce self-neglect.

Our results showed that the mice exposed to chronic corticosterone gained their weights possibly due to an increase in food intake and water consumption. This seems contradictory to previous findings that stressed animals lose body weight and clinical observations that depressive patients tend to lose weight. This discrepancy can be explained by the difference in the method of providing chronic stress. A previous study found increased body weight, increased plasma insulin and leptin levels and decreased insulin sensitivity after four-week corticosterone administration via drinking water, proposing a potential animal model of the metabolic syndrome [26]. There are also other studies reporting increased body weight by chronic corticosterone exposure [34,35]. Water consumption was also increased in mice exposed to chronic corticosterone [36]. Increased water consumption in this study cannot be explained by increased ethanol consumption because a previous study found that two-week intracerebral infusion of corticosterone did not increase ethanol intake in control rats [37]. These suggest that gain in body weights, increased food intake and water consumption are due to our method of chronic corticosterone administration.

What are the neural mechanisms underlying self-care behaviours in rodents? As for nesting behaviour, a previous study found a population of hippocampal neurons responsive to being in the nest [38]. Another study found that optogenetic stimulation of midbrain dopamine neurons promotes wakefulness and inhibits nesting behaviour [39]. As for self-grooming behaviours, studies have shown that several brain regions such as the basal ganglia, the amygdala and the hypothalamus are involved [18]. For example, *Sapap3*-mutant mice which have synaptic abnormality in cortico-striatal synapses exhibited abnormal self-grooming behaviour [40]. Although the defecation centre is thought to be located in the brainstem and in the spinal cord [41,42], the neural circuitry underlying latrine behaviour has been unclear. These results suggest that individual self-care behaviours may employ distinct neural circuits. It remains to be elucidated if there is a common upstream centre for self-care behaviours.

Excessive glucocorticoids are known to damage the nervous system and cause impairment across various brain functions [43,44]. Stressor information activates the hypothalamus, which in turn promotes the release of glucocorticoids from the adrenal cortex through activating the hypothalamic–pituitary–adrenal axis. In humans, patients with hypercortisolemia due to Cushing's syndrome show cognitive deficits and reduced hippocampal volume [45]. Patients with major depressive disorder, which is associated with an abnormal glucocorticoid system [43,46,47], also exhibit reduced hippocampal volume [48]. In rodent experiments, chronic corticosterone administration is known to cause morphological changes in several brain regions such as the hippocampus, the amygdala and the prefrontal cortex [25,49,50]. Furthermore, it is associated with behavioural changes such as increased depression-like behaviour [23,24], decreased spatial learning function [51] and increased fear response [52]. This subject has implications for future research in terms of how these neural changes are related to the maintenance of self-care behaviours.

Self-care behaviour refers to appropriately maintaining one's health and surrounding environment. This study shows that self-care behaviours can be examined experimentally by quantifying spontaneous home cage behaviours such as latrine and nesting behaviours. Thus, this study may provide a promising model for mechanistic studies of self-neglect. Recent studies have examined a wide variety of rodent behaviours, ranging from instinctive behaviour to cognitive behaviour, by using specific and elaborated behavioural settings [53–56]. In complement to discrete behavioural paradigms, studying home cage behaviours provides valuable insight into spontaneous behaviours such as self-care.

## Data Availability

Raw data is available via Dryad Digital Repository: https://doi.org/10.5061/dryad.ffbg79cxp [57]. The data are provided in the electronic supplementary material [58].
